# Quercetin Inhibits Intestinal Iron Absorption and Ferroportin Transporter Expression *In Vivo* and *In Vitro*

**DOI:** 10.1371/journal.pone.0102900

**Published:** 2014-07-24

**Authors:** Marija Lesjak, Rukshana Hoque, Sara Balesaria, Vernon Skinner, Edward S. Debnam, Surjit K. S. Srai, Paul A. Sharp

**Affiliations:** 1 Research Department of Structural and Molecular Biology, Division of Biosciences, University College London, London, United Kingdom; 2 Department of Chemistry, Biochemistry and Environmental Protection, Faculty of Sciences University of Novi Sad, Novi Sad, Serbia; 3 Diabetes & Nutritional Sciences Division, King's College London, London, United Kingdom; 4 Research Department of Neuroscience, Physiology and Pharmacology, Division of Biosciences, University College London, Royal Free Campus, London, United Kingdom; University of Florida, United States of America

## Abstract

Balancing systemic iron levels within narrow limits is critical for maintaining human health. There are no known pathways to eliminate excess iron from the body and therefore iron homeostasis is maintained by modifying dietary absorption so that it matches daily obligatory losses. Several dietary factors can modify iron absorption. Polyphenols are plentiful in human diet and many compounds, including quercetin – the most abundant dietary polyphenol – are potent iron chelators. The aim of this study was to investigate the acute and longer-term effects of quercetin on intestinal iron metabolism. Acute exposure of rat duodenal mucosa to quercetin increased apical iron uptake but decreased subsequent basolateral iron efflux into the circulation. Quercetin binds iron between its 3-hydroxyl and 4-carbonyl groups and methylation of the 3-hydroxyl group negated both the increase in apical uptake and the inhibition of basolateral iron release, suggesting that the acute effects of quercetin on iron transport were due to iron chelation. In longer-term studies, rats were administered quercetin by a single gavage and iron transporter expression measured 18 h later. Duodenal FPN expression was decreased in quercetin-treated rats. This effect was recapitulated in Caco-2 cells exposed to quercetin for 18 h. Reporter assays in Caco-2 cells indicated that repression of FPN by quercetin was not a transcriptional event but might be mediated by miRNA interaction with the FPN 3′UTR. Our study highlights a novel mechanism for the regulation of iron bioavailability by dietary polyphenols. Potentially, diets rich in polyphenols might be beneficial for patients groups at risk of iron loading by limiting the rate of intestinal iron absorption.

## Introduction

Iron is an essential transition metal that plays an important role in all mammalian organisms. It is incorporated into a diverse array of proteins, including the oxygen carriers haemoglobin and myoglobin, cytochrome complexes involved in electron transfer in the mitochondria, and enzymes participating in nucleic acid processing such as ribonucleotide reductase [1], [2]. Balancing systemic iron levels within narrow limits is critical for human health, as both iron deficiency and iron overload leads to serious haematological, metabolic and neurodegenerative disorders. In mammals there are no known pathways to eliminate excess iron from the body and therefore iron homeostasis is maintained by modifying dietary absorption so that it matches daily obligatory losses [1].

There are two forms of dietary iron, haem and non-haem iron. Non-haem iron is the major dietary form but its bioavailability depends on presence of other dietary factors which either enhance or inhibit absorption in the duodenum [3], [4]. Non-haem iron is present almost entirely in the ferric (Fe^3+^) form; however, to be bioavailable it must first be reduced to ferrous (Fe^2+^). This is achieved by the combined action of duodenal cytochrome b (Dcytb), a ferri-reductase which resides on the apical membrane of duodenal enterocytes [5], [6], or exogenous dietary reducing agents, such as ascorbic acid (reviewed in [4]). Reduced iron is then transported across the membrane into the enterocyte via the apical iron transporter, divalent metal transporter-1 (DMT1) [7]. Subsequently, ferrous iron is transferred across the basolateral membrane of the enterocyte via the iron exporter, ferroportin (FPN) [8]–[10] and re-oxidized by ferroxidase hephaestin [11] on the basolateral surface, prior to loading onto transferrin.

In addition to enhancers of iron bioavailability there are a number of dietary components that act as potent inhibitors of intestinal iron absorption, including phytic acid and polyphenolic compounds [4]. Polyphenols are natural products, which are abundant in food of plant origin, and are thus an integral part of our diet. Dietary polyphenols are receiving increasing attention due to their proven health benefits for a variety of disorders [12]. The inhibitory potential of flavonoid polyphenols on non-haem iron absorption in duodenum has been demonstrated both *in vivo* and *in vitro* [13]–[17]. These studies provide convincing evidence that polyphenols modify intestinal iron absorption in single meal studies in human volunteers and acute *in vitro* experiments; however, the longer-term effect of consuming elevated levels of polyphenolic compounds on iron status is less clear. It is possible that chronic consumption of diets poor in iron and rich in inhibitors of iron bioavailability could contribute to the burden of iron deficiency in certain population groups. However, there may be benefits of consuming a polyphenol-enriched diet for groups at risk of iron loading, for example patients with hereditary haemochromatosis.

The flavonol quercetin is the most abundant dietary flavonoid and is especially enriched in onions, tea and apples [18]. It is conservatively estimated that humans consume approximately 40 – 80 mg of flavonoids/day [19]; and that quercetin contributes approximately 25% of total flavonoid intake (i.e. 10–20 mg/day) [20]. In common with most polyphenols, quercetin is found almost exclusively in foods as glycoside conjugates but can be converted rapidly into the aglycone in the intestinal lumen via the actions of glycosidases [21], [22]. In this study we have investigated the acute and longer-term effects of quercetin on iron metabolism *in vivo* (in rats) and *in vitro* (Caco-2 cells).

## Material and Methods

### Animals and treatments

Rats were supplied by the Comparative Biology Unit, Royal Free Campus, UCL Medical School, London, UK. All experimental procedures were approved by the University College London local animal ethics committee and were conducted in accordance with the UK Animals (Scientific Procedures) Act, 1986. After weaning (three weeks old) Sprague-Dawley male rats were placed on low iron (25 ppm) diet for two weeks and allowed free access to water throughout. At the end of the experimental procedure animals were killed by administering a terminal dose of pentobarbitone sodium (120 mg/Kg body weight).

### Acute effects of quercetin on iron uptake

For acute studies, rats were anesthetized with 60 mg/Kg pentobarbitone sodium IP and 5 cm long segments of duodenum (starting 1 cm distal to the pylorus) were cannulated and rinsed free of their contents with warm saline (0.9% w/v of NaCl), followed by air. Uptake buffer (200 µL), containing Na-HEPES (14.6 mmol/L), NaCl (127.4 mmol/L), KCl (3.2 mmol/L), ascorbic acid (4.0 mmol/L), ^59^FeCl_3_ (0.2 mmol/L) and 1 mmol/L of either quercetin (Sigma-Aldrich, UK) (Q) or 3-*O*-methylquercetin (Extrasynthese, France) (3MQ) or 4′-*O*-methylquercetin (Extrasynthese) (4′MQ) or quercetin 3,4′-dimethylquercetin (ABCR GmbH, Germany) (3,4′dMQ) or penta-methylquercetin (Extrasynthese) (PMQ) (Stock solutions prepared in DMSO:ethanol [1∶1]) was instilled into the duodenal segment, which was then tied off. In control groups, DMSO:ethanol (1∶1) was added to buffer instead of the polyphenols. Structures of polyphenolic compounds are shown in Figure S1. Rat body temperature was maintained at 37°C using a thermostatically controlled heating blanket. After 30 minutes, blood samples (≤2 mL) were collected via cardiac puncture and the segment of duodenum was removed and washed with approximately 40 mL of solution containing 154 mmol/L NaCl, 0.1 mmol/L ascorbic acid, 0.01 mmol/L FeCl_3_ to displace any ^59^Fe bound to the mucosal surface. Subsequently, the duodenal mucosa was scraped away, the blood and mucosa samples were weighed and gamma counted for determination of ^59^Fe activity. Results were expressed as percentage of absorbed radioactive iron retained in duodenal mucosa or transferred to blood. The percentage of ^59^Fe transferred to the entire blood volume of the animal was calculated using the equation: total blood volume  =  (body weight*0.06) +0.77 [23].

### Effects of oral gavage of quercetin on iron metabolism

In order to investigate the longer-term effects of quercetin administration on non-haem iron absorption, animals were gavaged with a single dose of quercetin (50 mg/kg dissolved in 10% DMSO) or vehicle (control) eighteen hours before experiments. Animals were anesthetized with pentobarbitone sodium (60 mg/Kg body weight) injected IP. The duodenum and liver were collected, snap frozen in liquid N_2_, and stored at −80°C for real-time PCR analysis and tissue non-haem iron determination.

### RNA extraction and RT-PCR

Duodenal RNA was extracted with TRIzol (Invitrogen) according to the manufacturer's instructions. Total RNA (1 µg) was reverse transcribed using the Verso cDNA reverse transcription kit (Thermo-Fisher Scientific), according to the manufacturer's instructions. Real-time PCR reactions were performed using a Roche Lightcycler using GAPDH as internal standard. Each reaction was performed in duplicate and contained 10 pmol of specific primers (Table S1), 1× SYBR Green Mastermix (Qiagen), and 1 µL of cDNA in a 20 µL reaction. Samples without cDNA were included as negative controls. Cycle threshold (Ct) values were obtained for each gene of interest and the GAPDH internal standard. Gene expression was normalized to GAPDH and represented as ΔCt values. For each sample the mean of the ΔCt values was calculated. Relative gene expression was normalized to 1.0 (100%) of controls.

### Levels of tissue non-haem iron

Quantitative measurement of non-haem iron was performed according to the modified method of Torrance and Bothwell [24]. Briefly, 20–50 mg of duodenum was dried at 55°C for 72 hours and subsequently weighed. Dried samples were digested in 1 mL acid mixture (30% (v/v) HCl and 10% (w/v) trichloroacetic acid) at 65°C for 20 hours. 1 mL of chromogen reagent (0.1% (w/v) bathophenanthrolinesulfonate; 1% (v/v) thioglycolic acid in 50% (w/v) sodium acetate solution) and samples were incubated at 37°C for 10 minutes, after which the absorbance of samples and iron standards was measured at 535 nm. Results were reported as µg iron/g tissue dry weight.

### Serum biochemistry

Serum iron and transferrin saturation were measured using a commercial ferrozine-based colorimetric assay according to the manufacturer's instructions (Pointe Scientific, USA).

### Cell culture

Caco-2 TC7 cells were originally obtained from Drs Monique Rousset and Edith Brot-Laroche (CRC, Jussieu, Paris) [25] and have been characterised for studies on iron transport [26]. Cells (passages 40-45) were seeded onto Transwell inserts in 6-well plates at a density of 40,000 cells per well and cultured for 19 d in DMEM containing 10% foetal bovine serum.

For acute uptake studies, cells were pre-treated with quercetin (10 or 100 µmol/L) for 15 min to permit equilibration in uptake buffer; quercetin remained present during the uptake assay. For chronic studies, cells were exposed for 18 h at their apical surface to quercetin. However, prior to uptake measurements, the incubation medium was discarded and cells were washed to remove quercetin.

### In vitro iron transport

Iron uptake was measured as described previously [27]. Briefly, a transepithelial pH gradient was established by adding Mes-buffered saline (pH 5.5; 140 mmol/L NaCl, 5 mmol/L KCl, 1 mmol/L Na_2_HPO_4_, 1 mmol/L CaCl_2_, 0.5 mmol/L MgCl_2_, 5 mmol/L glucose, and 10 mmol/L Mes) and adding Hepes-buffered saline (pH 7.5; same ionic composition but containing 10 mmol/l Hepes in place of Mes and 0.2% bovine serum albumin) to the basolateral chamber. Studies were initiated by the addition of 10 µmol/L ^55^FeCl_3_ complexed with 1 mmol/L ascorbic acid to the apical well of the Transwell plate. Uptake into the cells was measured over 20 min. To measure iron efflux, cells were incubated for 1 h in buffer containing ^55^FeCl_3_, washed and placed in fresh medium, and incubated for a further 2 h at 37 C. At the end of the experiments cells were washed thoroughly in uptake buffer containing iron and solubilized in 0.2 mmol/L NaOH. Basolateral medium from efflux assays was collected and aliquots of the solubilized cell fraction and basolateral medium were subjected to liquid scintillation counting. Data were normalized to amounts of total protein/well.

### PCR

Total RNA was isolated from cells cultured for 18 h in the presence of quercetin using TRIzol. Following first strand cDNA synthesis, expression levels of iron transporter mRNA and GAPDH mRNA (used as a housekeeping gene) were analysed by real-time quantitative PCR using an ABI Prism 7700HT Sequence Detection System and a Power SYBR Green PCR master mix kit (Applied Biosystems Cheshire, UK). The primer sequences used for each gene are given in Table S1. Quantitative measurements of iron transporter relative to GAPDH gene expression were derived using the ΔCt method. Data have been normalised to the untreated control group in each experiment and are presented as the mean ± S.E.M.

### Western blotting

Caco-2 cells were harvested into ice-cold lysis buffer (PBS containing 1% sodium dodecyl sulphate and 10 mg/L protease inhibitor cocktail) and homogenized by repeated passing through at 25-gauge needle. Protein samples (40 µg) were solubilised in sample loading buffer and subjected to polyacrylamide gel electrophoresis. Following immobilization on nitrocellulose, the proteins were exposed to commercially available antibodies to either DMT1 (NRAMP24A; 1∶1000 dilution, Alpha Diagnostics, TX, USA), FPN (MTP11A, 1∶1000 dilution, Alpha Diagnostics) or β-actin (1∶1000 dilution, Sigma-Aldrich). Cross-reactivity was observed using a horseradish peroxidase-linked secondary antibody (Dako) and ECL Plus (GE Healthcare).

### Cell ferritin levels

Cells were incubated with 10 µmol/L FeCl_3_ in the presence or absence of quercetin (0.1–10 µmol/L) for 18 h. Cells were washed with PBS before being lysed in ice-cold lysis buffer (10 mmol/L Tris-HCl, pH 7.5, 50 mmol/L NaCl, 1% Triton X-100, 10 mg/L protease inhibitor cocktail). 20 µg of cellular protein was used to measure cell ferritin using a commercially available ELISA kit (RAMCO Laboratories).

### Reporter assays

FPN 5′-promoter-luciferase constructs were provided by Drs D-L Zhang and T. Rouault (National Institute of Child Health and Human Development, USA). MISSION FPN 3′-UTR-Renilla luciferase reporter construct was purchased from Sigma-Aldrich UK. Caco-2 cells were seeded in 24-well plates and grown to 60-80% confluence. Cells were transfected with reporter constructs and control plasmids using FuGENE HD transfection reagent (Promega UK). After 24 h, cells were treated with quercetin (10 µmol/L) for 18 h and luciferase activity measured (in duplicate, in 3 plates of cells, repeated in at least 2 individual passages; n = 6–9) using a Promega dual-luciferase reporter assay system.

### Statistics

All data are expressed as means + SEM. Statistical analysis was carried out using SigmaPlot (version 12, Systat Software Inc. IL, USA). Student's unpaired t-test or one-way ANOVA followed by Tukey's post-hoc test were used where appropriate to detect statistical differences (P<0.05) between control and test groups.

## Results

### Acute exposure to quercetin increases apical iron uptake but decreases basolateral iron efflux

**Figure 1:**
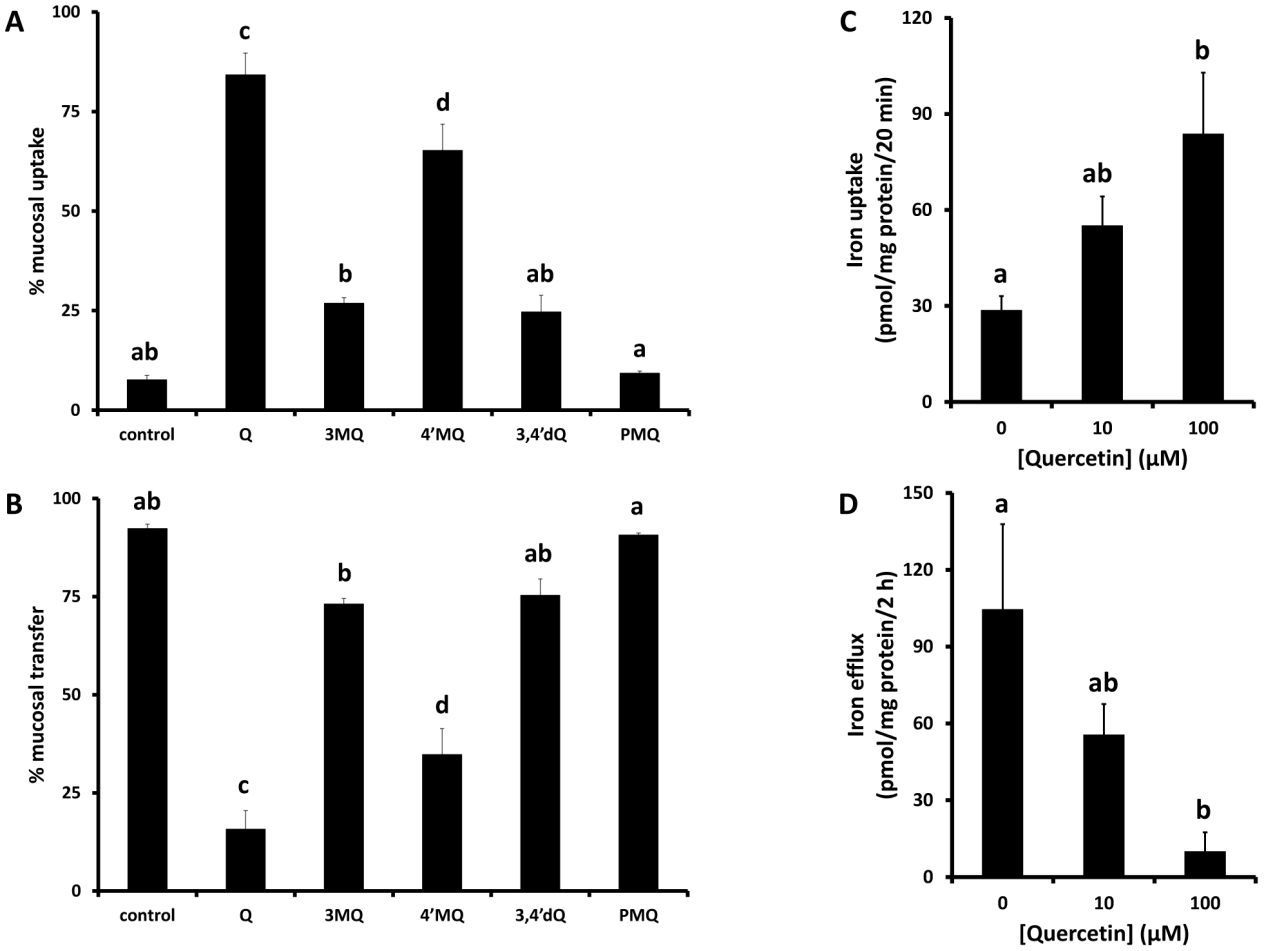
Acute effects of quercetin on intestinal iron transport. The acute effects of quercetin and its methylated analogues (3MQ, 4′MQ, 3,4′dMQ, PMQ) on iron transport *in vivo* were measured using the *in situ* duodenal loop method. ^59^Fe (0.2 mmol/L) and polyphenol (1 mmol/L) were added to the lumenal uptake buffer and mucosal iron uptake (A) and mucosal transfer of iron into the circulation (B) measured. The effects of quercetin on iron uptake across the apical membrane (C) and iron efflux across the basolateral membrane (D) of human intestinal Caco-2 cells was also measured. Data are mean ± SEM of 4 (rat duodenum) or 4–5 (Caco-2 cells) observations in each group. Groups with no common letters are significantly different from each other (one-way ANOVA and Tukey's post hoc test; P<0.05).

To investigate the acute effects of quercetin on duodenal iron absorption, the *in situ* duodenal loop method was used [28] where either quercetin (aglycone) or methylated quercetin isoforms were introduced into rat duodenum together with radioactive iron. In the presence of quercetin, 3MQ, 4′MQ or 3,4′dMQ, but not PMQ, there was a significant increase in mucosal ^59^Fe uptake compared with the untreated control group (Figure 1A). The increase in uptake was significantly higher in the presence of quercetin aglycone and 4′MQ compared with the other methylated forms. In contrast, ^59^Fe release from the intestinal mucosa into the blood was significantly diminished in the presence of quercetin and 4′MQ compared with other methylated-quercetin compounds and the untreated controls (Figure 1B).

The acute stimulation of iron uptake as well as inhibition of iron efflux in the presence of quercetin aglycone was recapitulated in intestinal Caco-2 cell monolayers (Figure 1C,D).

### Quercetin decreases intestinal FPN expression

**Figure 2:**
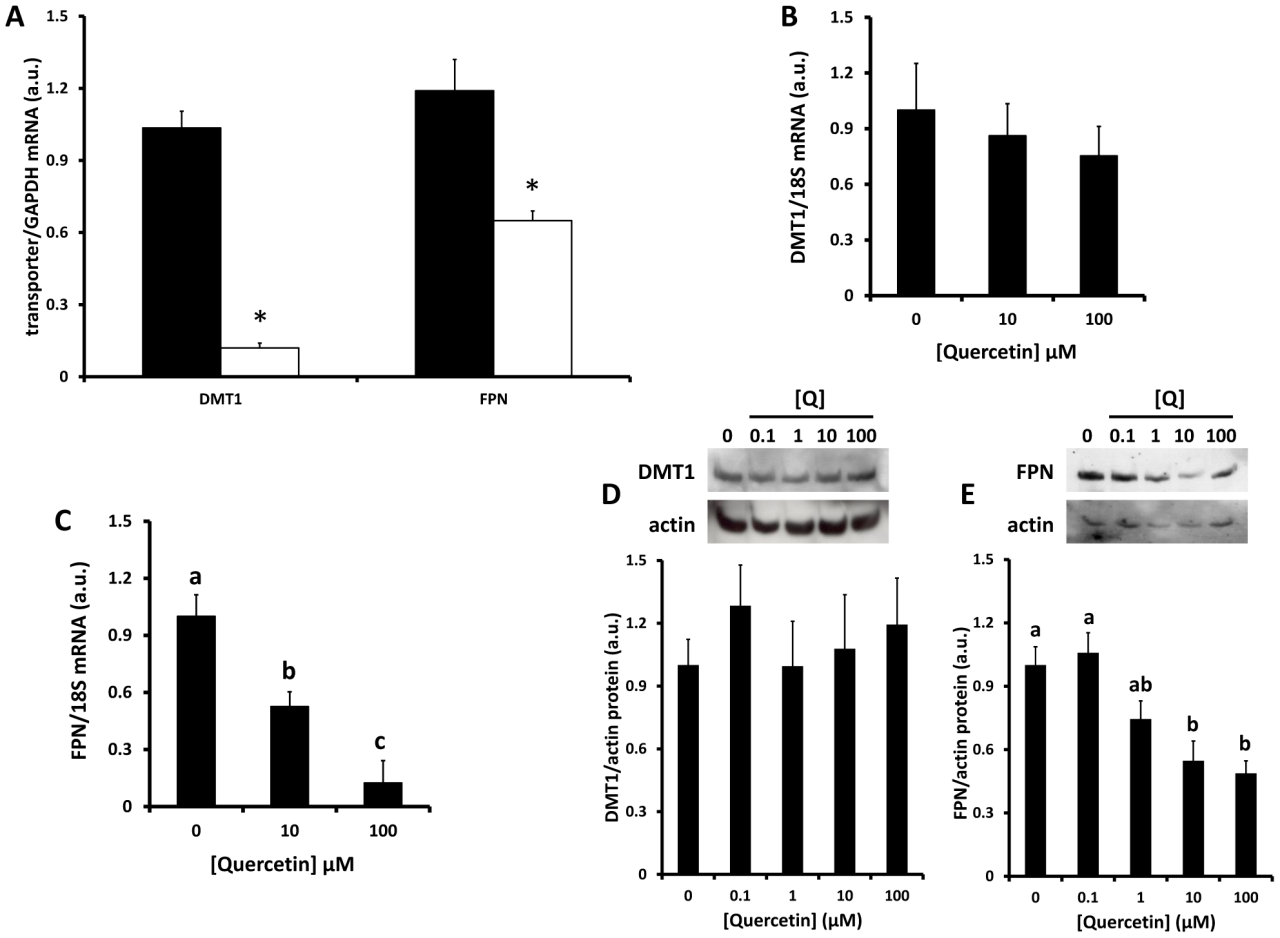
Effects of longer-term exposure to quercetin on intestinal iron transporter expression. Rats (A) were given a single gavage containing quercetin (50 mg/Kg body weight). Caco-2 cells (B & C) were exposed to quercetin (10 or 100 µM). After 18 h RNA was isolated from duodenal tissue (A) and Caco-2 cells (B,C) and used to measure changes in iron transporter mRNA expression. (D,E) Whole cell protein was isolated from Caco-2 cells and used for western blotting to measure DMT1 and FPN protein. Data are mean ± SEM of 4 (rat duodenum) or 7-18 (Caco-2 cells) observations in each group. (A) *P<0.05 compared with control group (Student's unpaired t-test). (B – E) Groups with no common letters are significantly different from each other (one-way ANOVA and Tukey's post hoc test; P<0.05). No letters indicates no significant difference between any of the groups.

18 hours after oral administration of a single dose of quercetin, quantitative RT-PCR analysis of rat duodenal iron transporters revealed significant down regulation of DMT1 and FPN mRNA levels (Figure 2A). In the same animals there was a significant increase in non-haem iron content of the duodenum, a significant decrease in serum iron and transferrin saturation and a decrease in liver hepcidin mRNA expression (60%) which did not reach statistical significance compared with control animals (Table 1).

**Table 1:** Markers of iron status in control and quercetin-treated rats.

	Control	Quercetin-treated
Duodenal non-haem iron (µg/g dry weight)	22.9±1.2 (4)	32.5±1.5 (4)*
Serum iron (µg/dL)	140.9±15.1 (4)	90.7±3.9 (4)*
Transferrin saturation (%)	27.6±2.5 (4)	18.9±1.0 (4)*
Liver hepcidin mRNA (a.u.)	0.6±0.3 (4)	0.2±0.1 (4)^NS^

Data are means ± SEM (n). *P<0.05 compared with control group. ^NS^ Not significantly different from control group.

To distinguish between the direct effects of quercetin on iron transporter expression, and possible indirect influences resulting from either increased duodenal iron content or changes in hepcidin levels, we employed Caco-2 cells as a model of human intestinal epithelial cells. In contrast to data from rat studies, there was no effect of quercetin on DMT1 mRNA or protein levels in Caco-2 cells (Figure 2B,D). However, FPN mRNA and protein expression were decreased significantly by exposure to quercetin in a dose-dependent manner (Figure 2C,E).

**Figure 3:**
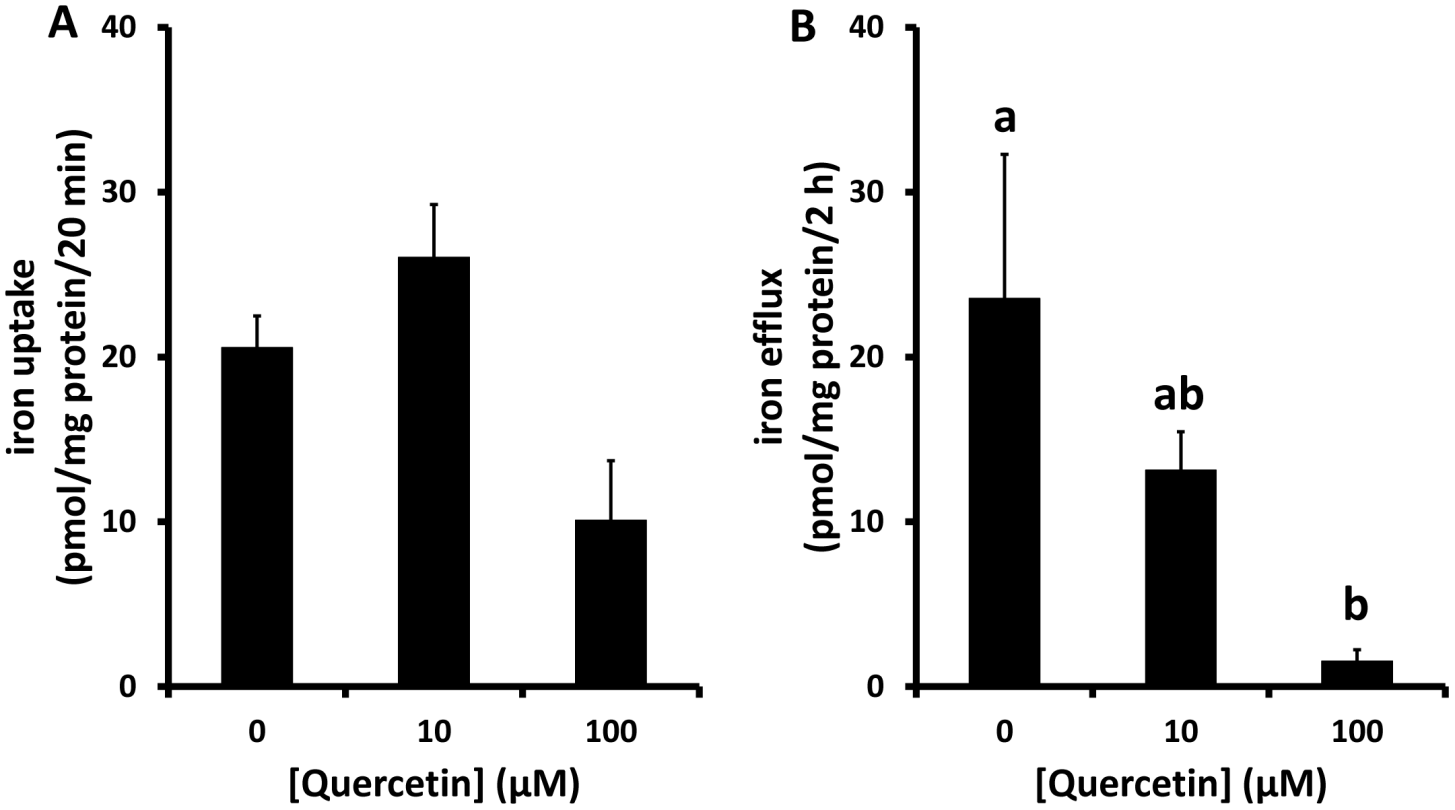
Effects of longer-term exposure to quercetin on iron transport in Caco-2 cells. Caco-2 cells were exposed to quercetin (10 or 100 µM) for 18 h. Quercetin was removed by washing and cells were incubated in quercetin-free uptake buffer containing ^59^Fe. Uptake across the apical membrane (A) and efflux across the basolateral membrane (B) was determined. Data are mean ± SEM of 5-6 observations in each group. Groups with no common letters are significantly different from each other (one-way ANOVA and Tukey's post hoc test; P<0.05). No letters indicates no significant difference between any of the groups.

To determine the cellular effects of quercetin on iron transport, Caco-2 cells were washed to remove any residual lumenal quercetin and placed in fresh quercetin-free uptake buffer prior to measurement of iron transport. The initial rate of iron uptake into cells was not significantly altered following quercetin treatment (Figure 3A). However, there was a significant decrease in iron efflux into the basolateral medium in cells exposed to quercetin (Figure 3B).

**Figure 4:**
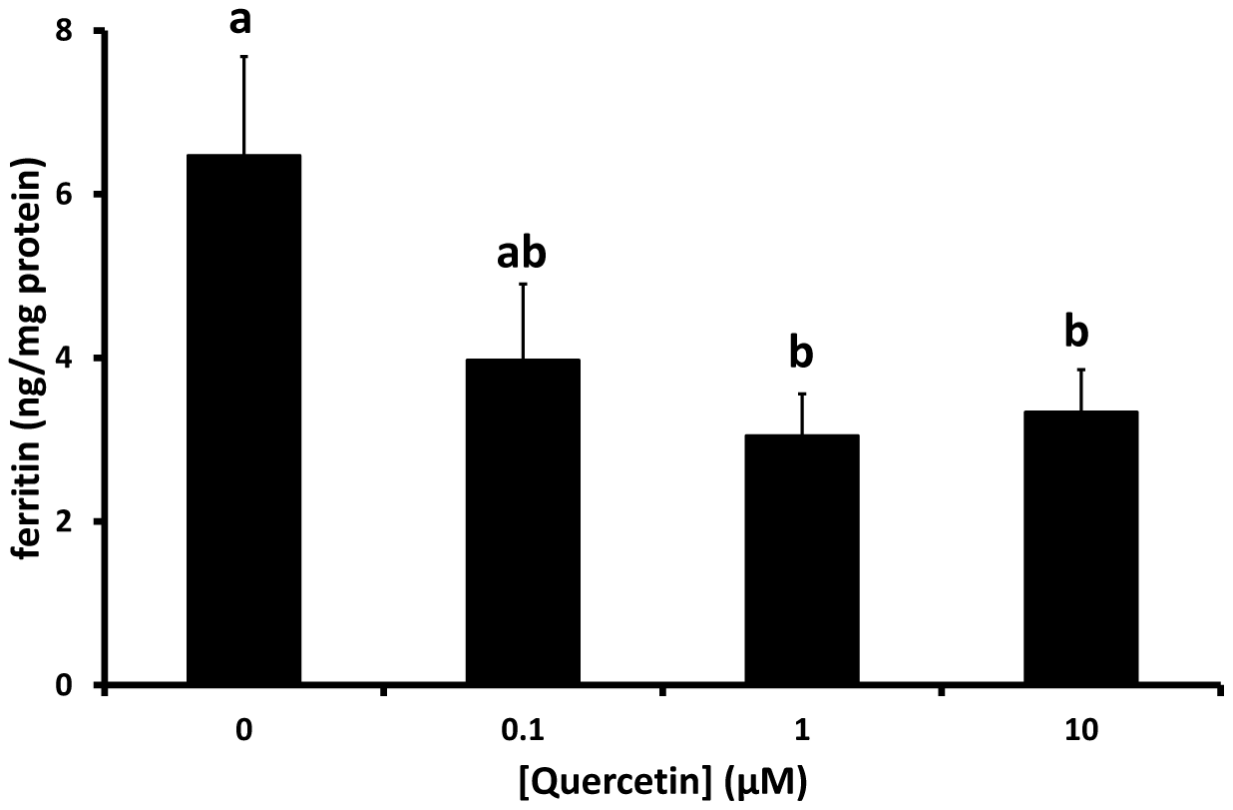
Effects of quercetin on ferritin protein levels in Caco-2 cells. Caco-2 cells were exposed to quercetin (10 or 100 µM) for 18 h. Cells were solubilised and aqueous extracts used to measured cell ferritin protein content using a commercially available ELISA. Data are mean ± SEM of 15 observations in each group. Groups with no common letters are significantly different from each other (one-way ANOVA and Tukey's post hoc test; P<0.05).

To investigate the cellular fate of cellular iron in quercetin-treated Caco-2 cells we measured cell ferritin content (the main cellular iron storage molecule). Following exposure to quercetin there was a significant decrease in ferritin protein levels (Figure 4).

### Quercetin did not alter FPN 5′-promoter activity in Caco-2 cells

**Figure 5:**
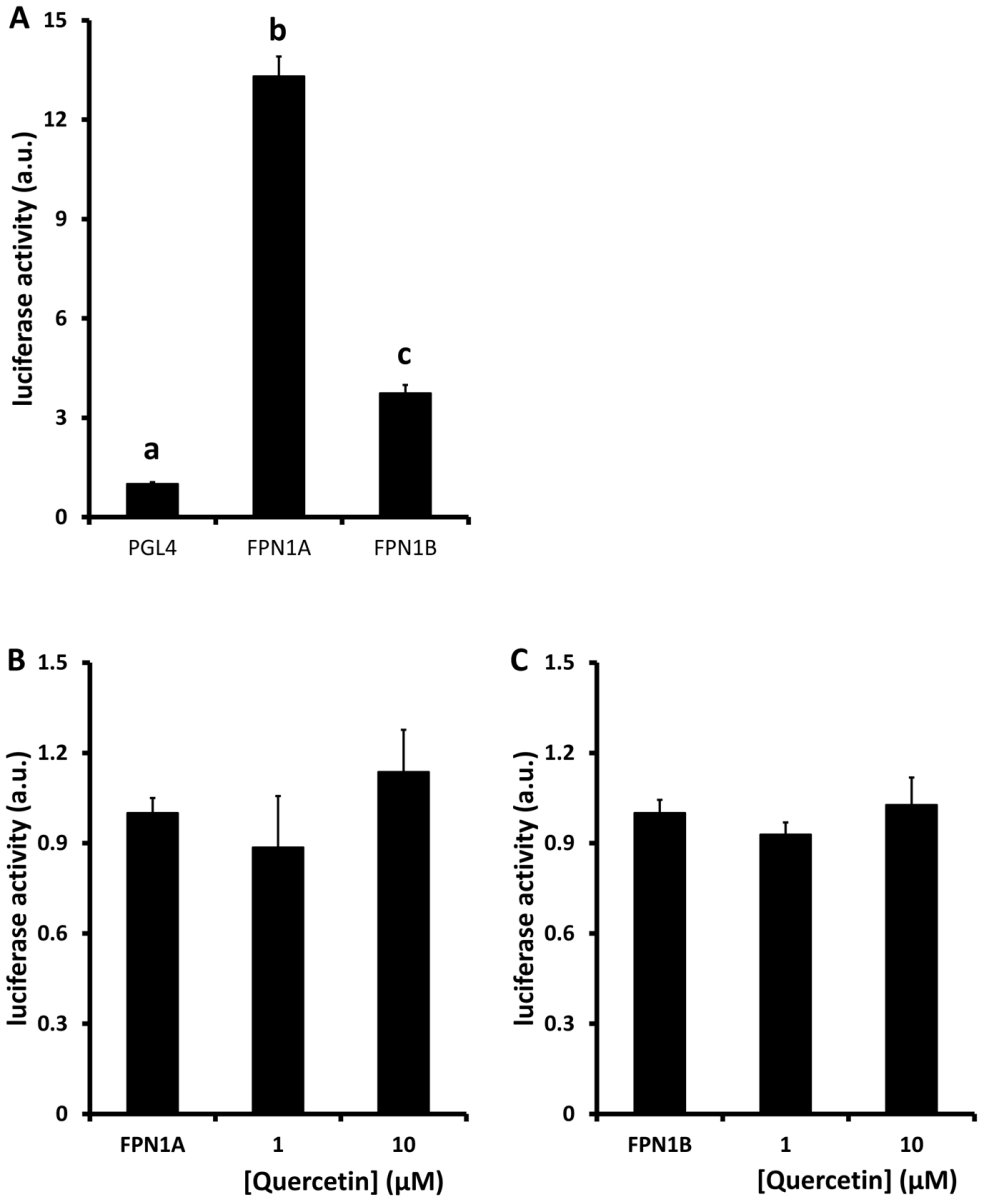
Effects of quercetin on FPN promoter activity. Caco-2 cells were transfected with luciferase reporter constructs containing 1Kb of either FPN1A or FPN1B 5′ promoter, or with the empty PGL4 plasmid. Luciferase activity (normalised to renilla) arising from basal activity of the promoters (A) and following exposure to quercetin (1 or 10 µM) for 18 h (B) was measured using the Dual Luciferase Reporter Assay (Promega). Data are mean ± SEM of 6-9 observations in each group. Groups with no common letters are significantly different from each other (one-way ANOVA and Tukey's post hoc test; P<0.05). No letters indicates no significant difference between any of the groups.

Next we investigated whether the inhibitory effects of quercetin on FPN protein and mRNA were mediate via altered gene transcription. Two FPN mRNA isoforms have been identified in Caco-2 cells; FPN1A and FPN1B, respectively [29]. Reporter assays with luciferase reporter constructs containing 1Kb of either the FPN1A or FPN1B 5′-promoter indicated that FPN1A is the dominant isoform in Caco-2 cells having 4-5 fold higher basal activity than the FPN1B promoter (Figure 5A). However, there was no effect of quercetin on the luciferase activity of either FPN promoter construct (Figure 5 B,C).

### Quercetin decreases 3′-UTR reporter activity in Caco-2 cells

**Figure 6:**
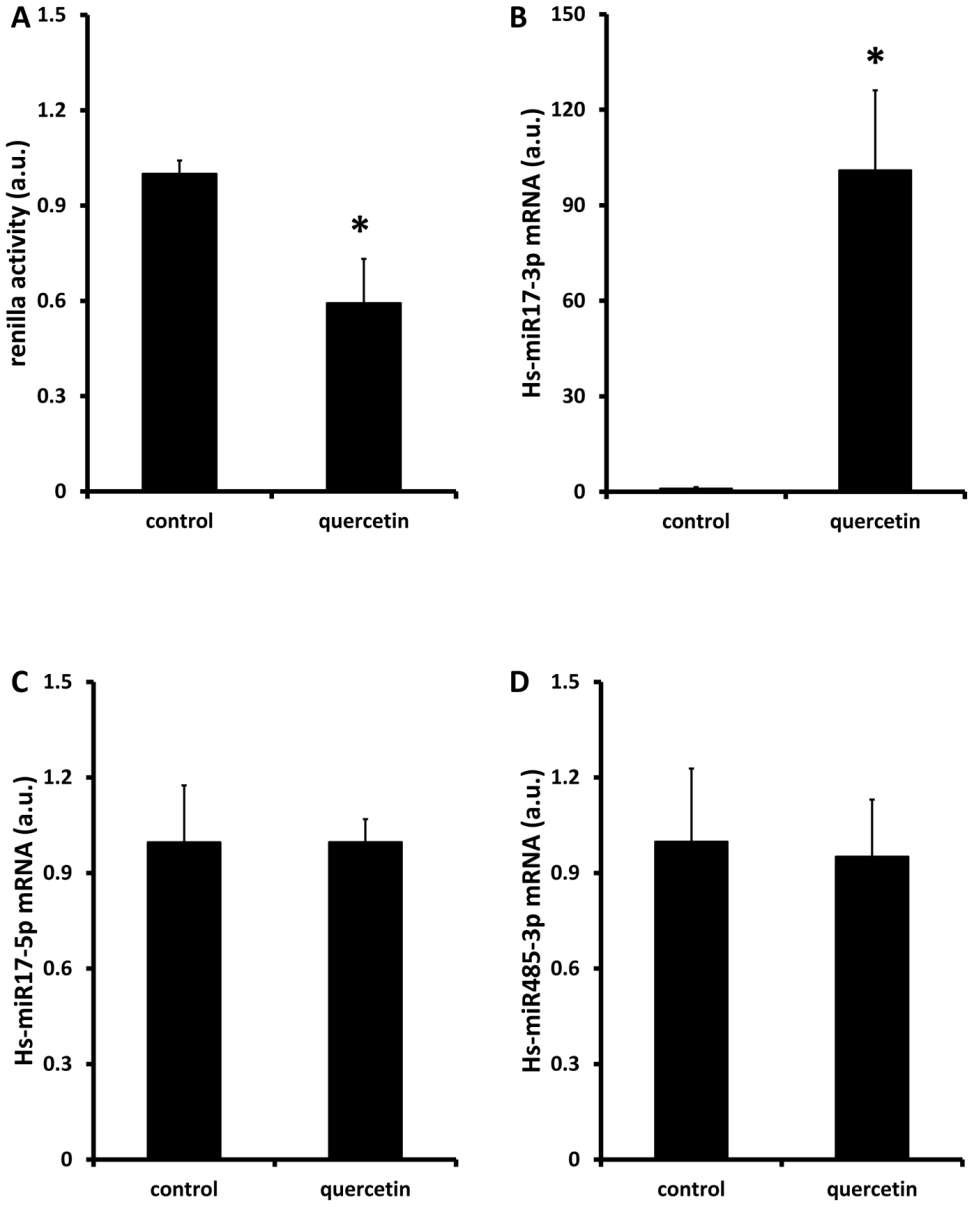
Effects of quercetin on the activity of FPN 3 ′ **UTR.** (A) Caco-2 cells were transfected with MISSION FPN 3′-UTR-Renilla luciferase reporter construct and exposed to quercetin (10 µM) for 18 h. (B-D) Quantitative PCR was used to determine the effects of quercetin on the expression of candidate microRNAs predicted to interact with the FPN 3′UTR. Data are mean ± SEM of 5-6 observations in each group. *P<0.05 compared with control group (Student's unpaired t-test).

Polyphenols are known to be potent regulators of micro-RNA (miRNA) expression and activity. We therefore investigated whether quercetin might regulate FPN mRNA levels in Caco-2 cells via miRNA interaction with its 3′UTR. In reporter assays in Caco-2 cells transfected with a Renilla-luciferase reporter construct containing the 3′ UTR of human FPN, exposure to quercetin significantly decreased reporter activity (Figure 6A).

Next we attempted to identify potential miRNA species that might be involved in these regulatory events. First, we used arrays to identify miRNA species up-regulated by quercetin in Caco-2 cells. Expression of 33 miRNAs were increased >1.5-fold in quercetin-treated cells compared with untreated controls (Table S2). Next we used web-based bioinformatics packages (TargetScan www.targetscan.org; and miRBase www.mirbase.org) to determine if any up-regulated miRNAs had consensus binding sites in the human FPN 3′-UTR (Table S3). Binding sites for 2 members of the miR-17 family, miR-17-3p (bases 61-67 of the 3′UTR sequence) and miR-17-5p (2 sites at bases 1132-1138 and 1166-1172), respectively, were present in the FPN 3′UTR (Figure S2). Finally, we used qPCR to validate the array data to determine whether expression of the miR-17 species was altered following quercetin treatment. miR-17-3p was strongly up-regulated in quercetin-treated Caco-2 cells (Figure 6B). In contrast, there was no significant effect of quercetin on the expression of miR-17-5p (Figure 6C). Furthermore, expression levels of miR-485-3p, which has 2 consensus binding sites in the FPN 3′UTR (at bases 448-454 and 618–625, respectively) and has been shown to be involved in the inhibition of FPN expression by iron deficiency in HepG2 cells [30], were not altered in quercetin-treated Caco-2 cells (Figure 6D).

## Discussion

Several independent studies have established that polyphenols significantly decrease non-haem iron absorption in duodenum [13]–[17]; however, the exact mechanism of action is unknown. Based on the data from our current study we discuss four possible mechanisms that might underlie the inhibitory effects of quercetin on intestinal iron transport: 1. Iron chelation; 2. Circulating iron and hepcidin levels; 3. Cellular iron levels; 4. Direct effects of quercetin on intestinal iron transporter expression.

### 1. Iron chelation

Our acute *in vivo* iron transport data demonstrated that quercetin increases iron uptake and retention by the duodenal mucosa. We argued that iron chelation by quercetin could play important role in this phenomenon. It has previously been shown that the preferred site for iron chelation by quercetin is between the 3-hydroxyl and 4-carbonyl group; for complexes containing one iron and one quercetin molecule, binding strength has an order 3–4>4–5>3′–4′. Moreover, 3–4 chelation site is also preferred for complexes which are formed between one iron and two or three quercetin molecules [31]. We therefore performed uptake experiments with quercetin aglycone, and methylated forms of quercetin to determine whether replacing the putative iron binding groups would influence transepithelial iron transport. Our data clearly indicate that the greatest increase in transepithelial iron transport was observed with compounds where 3-hydroxyl groups were methylated (3MQ, 3,4′dMQ and PMQ). In contrast, when the 3-hydroxyl group was present, namely in quercetin and 4MQ, there was a decrease in transepithelial iron transport. These results demonstrate that chelation of iron by the 3-hydroxyl group of quercetin is an important determinate of iron uptake in duodenum. Potentially this mode of action could be ascribed to all dietary polyphenols that have demonstrable capacity to chelate iron, for example (-)-epigallocatechin-3-gallate, [16], [32] and this information could be useful in the design of iron chelators based on the structure of these common dietary polyphenols.

The site of iron chelation by quercetin is unclear, i.e. whether binding takes place in the intestinal lumen or within the cytosol of the duodenal enterocytes. Quercetin aglycone can cross cell membranes via facilitated glucose (GLUT) transporters [33], [34]. In addition, there is evidence from *in vitro* studies that quercetin-iron chelates may also be transported via GLUTs [35]. Thus there is scope for both lumenal and cytosolic iron chelation to lead to iron accumulation within enterocytes. The increase in duodenal iron content occurs largely as a consequence of a reduced rate of iron efflux across the basolateral membrane into the blood. It is possible that intracellular quercetin-iron chelates might be too large to exit enterocytes via FPN, though some efflux through basolateral GLUTs remains a possibility. Furthermore, we cannot discount the possibility that quercetin or its metabolites have direct inhibitory effects on the function of FPN. Together these mechanisms could account for the increased mucosal iron retention observed in our studies.

### 2. Circulating iron and hepcidin levels

The longer term effects of quercetin on intestinal iron transport are less well studied. Here we have shown that oral administration of quercetin increased iron retention within the duodenum and resulted in a decrease in serum iron and transferrin saturation levels. In addition, there was a 60% decrease in liver hepcidin mRNA expression in quercetin-treated rats. These findings are consistent with previous data in rats fed an iron deficient diet [36]; however, crucially in this and previous studies iron deficiency was associated with a significant increase in both DMT1 [36], [37] and FPN in the duodenum [10], [36]. Furthermore, we and others have shown previously that intestinal FPN and DMT1 expression are down-regulated by prolonged exposure to elevated hepcidin levels [38]–[42]. Taken together these data suggest that low circulating iron and hepcidin levels do not mediate the inhibitory effects of quercetin on intestinal iron transport observed in the current study.

### 3. Cellular iron levels

The accumulation of iron in duodenal tissue observed in our *in vivo* experiments with quercetin-fed rats might also result in regulation of iron transporter expression. Previous studies in animals given a bolus of iron by gavage demonstrated a decrease in DMT1 expression [43]–[45]; however, iron efflux [43] and FPN expression [45] were largely unaffected. Therefore, the increase in duodenal iron accumulation in quercetin-fed rats is consistent with the decrease in DMT1 expression but does not explain the decrease in FPN.

The increase in duodenal iron content presents an apparent paradox since in quercetin-treated Caco-2 cells there was a significant decrease in levels of the iron storage protein ferritin, which is indicative of functional cellular iron deficiency rather than iron loading. In the cell culture model we envisage that cellular iron is sequestered by some other molecular entity, most likely quercetin. At this stage we do not know whether the iron retained within the rat duodenal mucosa in our *in vivo* model is also chelated by quercetin.

Even if we assume that quercetin treatment results in iron deficiency in Caco-2 cells this still cannot explain the effects of quercetin on transporter expression. We have shown that DMT1 expression in Caco-2 cells is decrease by iron [26]; [46] and increased following exposure to the iron chelator desferrioxamine (Figure S3). In contrast neither iron deficiency nor iron loading had a significant effect on FPN mRNA expression.

### 4. Direct effect of quercetin on intestinal FPN expression

Finally, we addressed the possibility that quercetin directly regulates FPN expression. In Caco-2 cells exposed to quercetin there was a significant dose-dependent decrease in FPN protein and mRNA and this was associated with a significant decrease in iron transport across Caco-2 cell monolayers. To elucidate the molecular mechanisms underlying these effects we employed luciferase-reporter assays containing either the FPN 5′-promoter or its 3′UTR. While there was no effect of quercetin on the activity of 5′-promoter, the activity of the 3′UTR construct was significantly decreased in the presence of quercetin. Regulation of the stability of the 3′UTR of mammalian mRNA has been shown to be controlled by a number of mechanisms including interactions with non-coding miRNA species. Interestingly, miRNAs have been shown to play a role in modulating the expression of a number of key players in iron homeostatic regulation, including hepcidin (miR-122; [47]), DMT1 (Let-7d; [48]) and FPN (miR-485-3p; [30]). While there was no significant effect of quercetin on miR-485-3p expression in Caco-2 cells, we identified miR-17-3p as a quercetin-induced candidate regulator of FPN mRNA expression. Expression of miR-17-3p was elevated significantly following exposure to quercetin, and bioinformatics identified a consensus binding site for miR-17-3p (bases 61-67 of the 3′UTR sequence) in the human FPN 3′UTR. The miR17 cluster is one of the most widely studied in microRNA biology and has been shown to play an important role in a number of biological processes in both health and disease [49]. Our data suggest a role for miR-17 and potentially other microRNAs in mediating diet-gene interactions that can influence nutrient bioavailability.

In summary, we have shown that quercetin can influence intestinal iron absorption acutely through chelation of iron within the intestinal lumen via its 3-hydroxyl group, and in the longer-term through direct regulation of FPN transporter expression, possibly through interactions between miRNA and 3′UTR of FPN mRNA. As potent inhibitors of iron bioavailability, diets rich in polyphenols might be beneficial for groups at risk of iron loading (e.g. patients with hereditary haemochromatosis), either by limiting the rate of intestinal iron absorption or by modifying tissue iron distribution. Further testing of quercetin and other phenolic compounds with iron chelating and cell signalling properties in animal models of iron overload could provide the basis for novel approaches for treating clinical iron overload in humans.

## Supporting Information

Figure S1 Structures of quercetin and methylated-quercetin analogues.
(TIF)

Figure S2 Predicted binding sites for miR17-3p, miR17-5p and miR485-3p in human FPN 3′UTR.
(TIF)

Figure S3 Effects of desferrioxamine (DFO) and iron (FAC) on (A) DMT1 and (B) FPN mRNA expression in Caco-2 cells. Data are mean ± SEM of 5–6 observations in each group. Groups with no common letters are significantly different from each other (one-way ANOVA and Tukey's post hoc test; P<0.05). No letters indicates no significant difference between any of the groups.
(TIF)

Table S1 PCR primers used in the study.
(TIF)

Table S2 Fold changes (>1.5-fold) of human miRNA in Caco-2 cells exposed to 10 µM quercetin for 18 h. miRNA profiling was performed using the GeneChip miRNA array (Affymetrix, UK), which contains 847 specific probe sets for human miRNAs derived from the Sanger miRBase miRNA database v11. Results from the chip have been filtered to show human miRNA only and true significant changes (p<0.05) when compared to untreated controls.
(TIF)

Table S3 Predicted miRNAs interacting with FPN (SLC40A1); top 30 by rank. Data are from miRBase www.mirbase.org. Both miR17-3p (24^th^) and miR17-5p (12^th^) are among the top 30 miRNAs predicted to interact with the FPN 3′UTR.
(TIF)
