# Supplementary figures and images for: Quercetin Inhibits Intestinal Iron Absorption and Ferroportin Transporter Expression In Vivo and In Vitro
Source: PLoS One. 2014 Jul 24;9(7):e102900. doi: 10.1371/journal.pone.0102900 (PMC4109952; doi:10.1371/journal.pone.0102900)

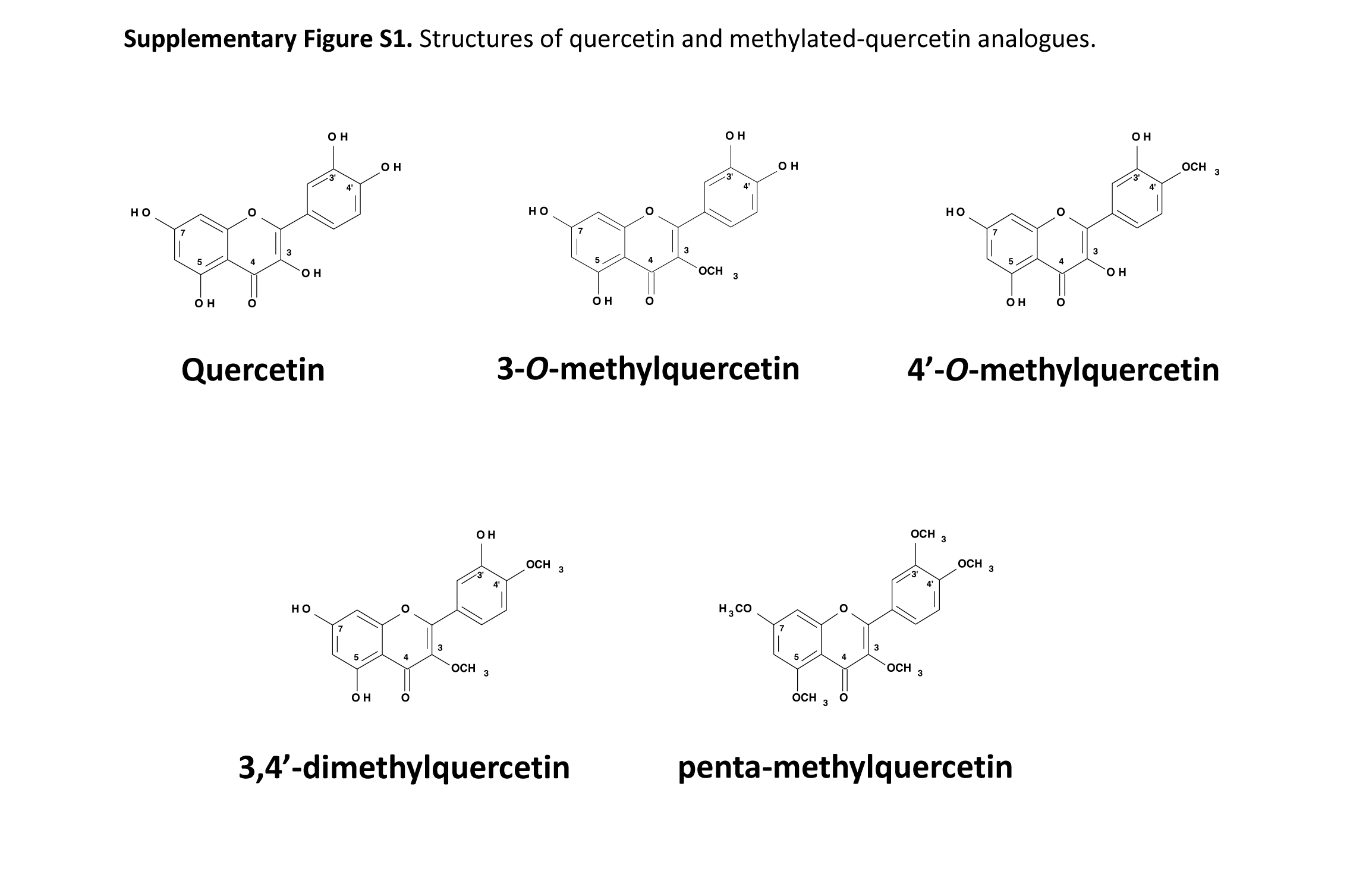

Supplement: Figure S1 — Structures of quercetin and methylated-quercetin analogues. (TIF) [file pone.0102900.s001.tif]

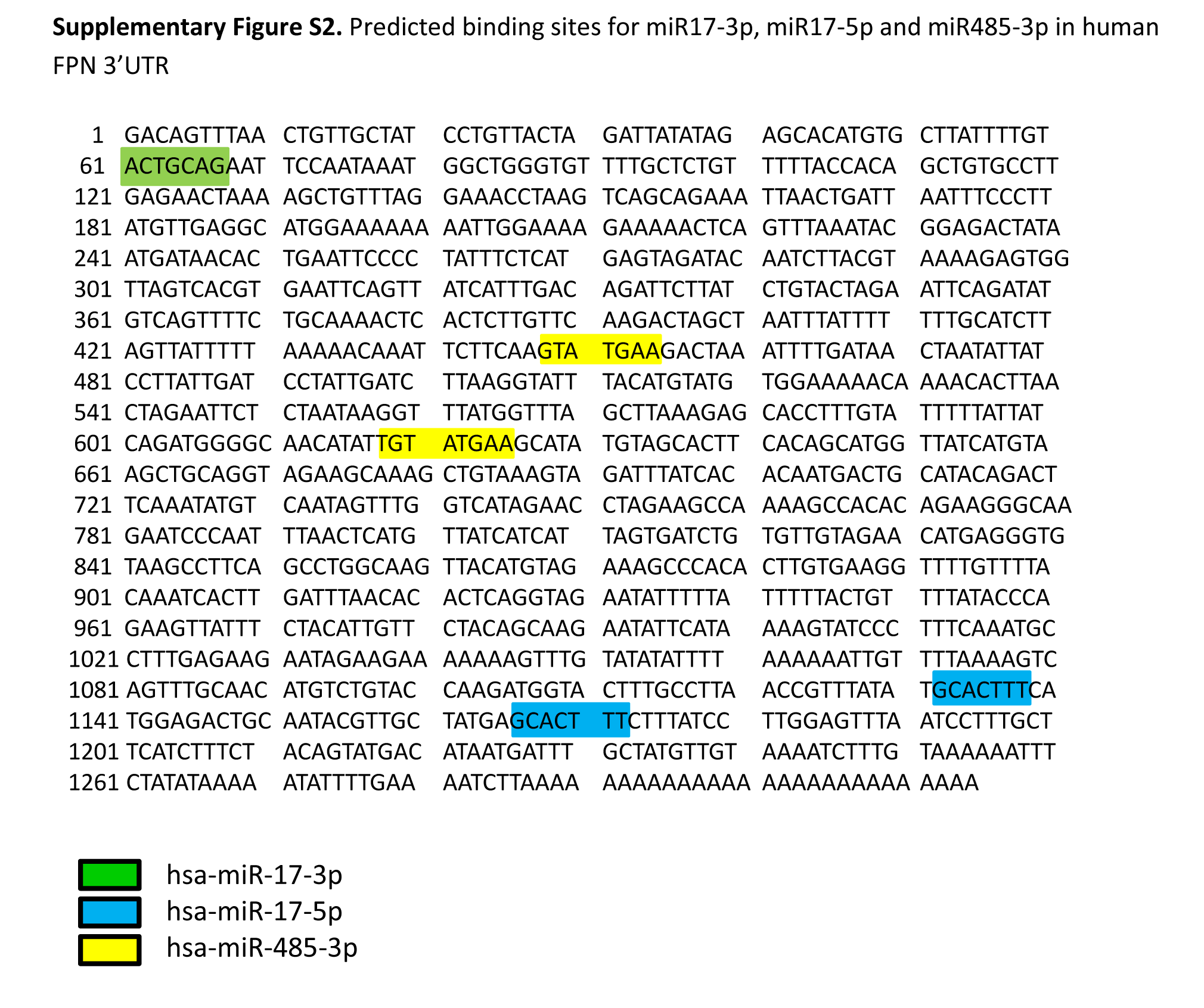

Supplement: Figure S2 — Predicted binding sites for miR17-3p, miR17-5p and miR485-3p in human FPN 3′UTR. (TIF) [file pone.0102900.s002.tif]

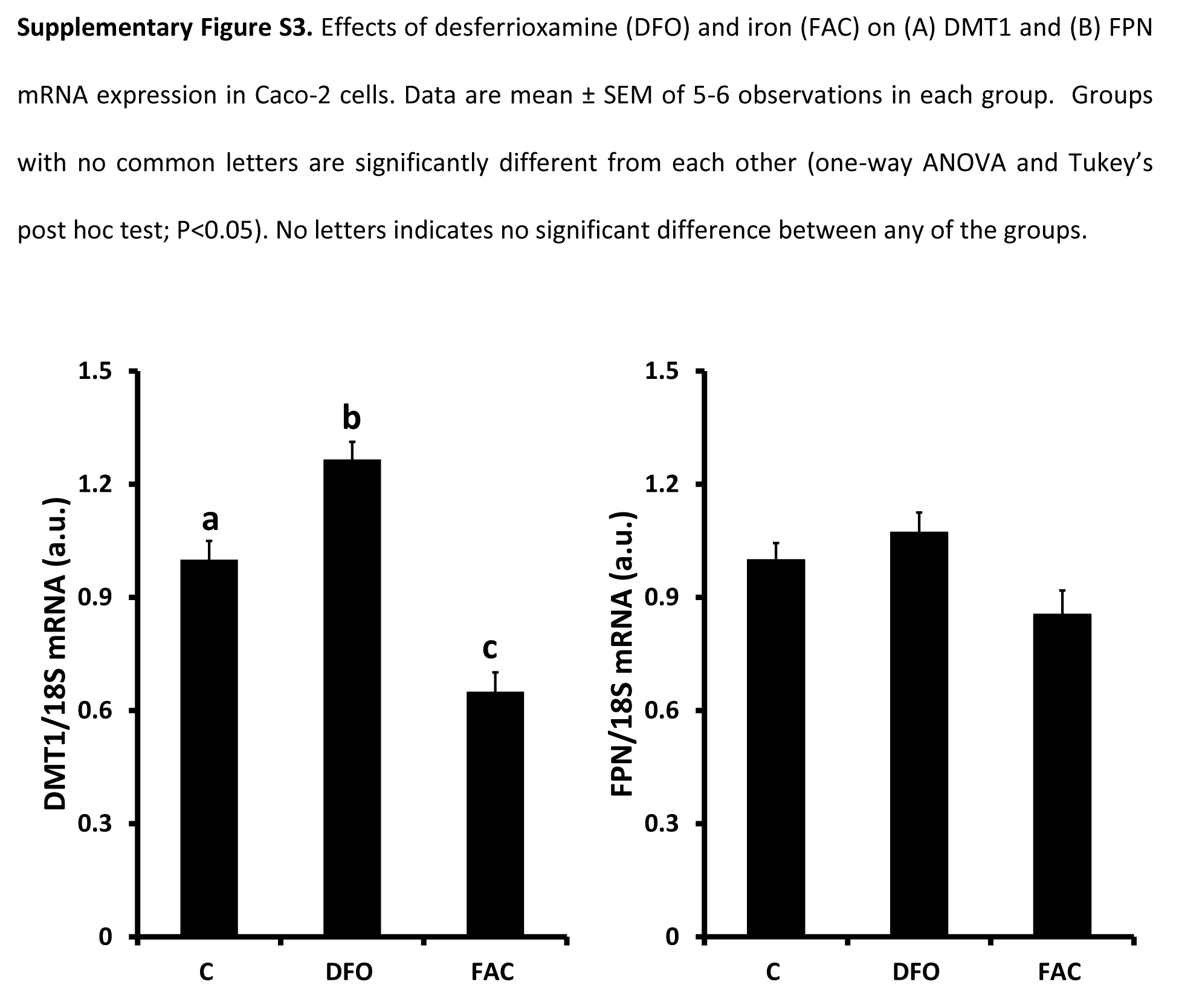

Supplement: Figure S3 — Effects of desferrioxamine (DFO) and iron (FAC) on (A) DMT1 and (B) FPN mRNA expression in Caco-2 cells. Data are mean ± SEM of 5–6 observations in each group. Groups with no common letters are significantly different from each other (one-way ANOVA and Tukey's post hoc test; P<0.05). No letters indicates no significant difference between any of the groups. (TIF) [file pone.0102900.s003.tif]

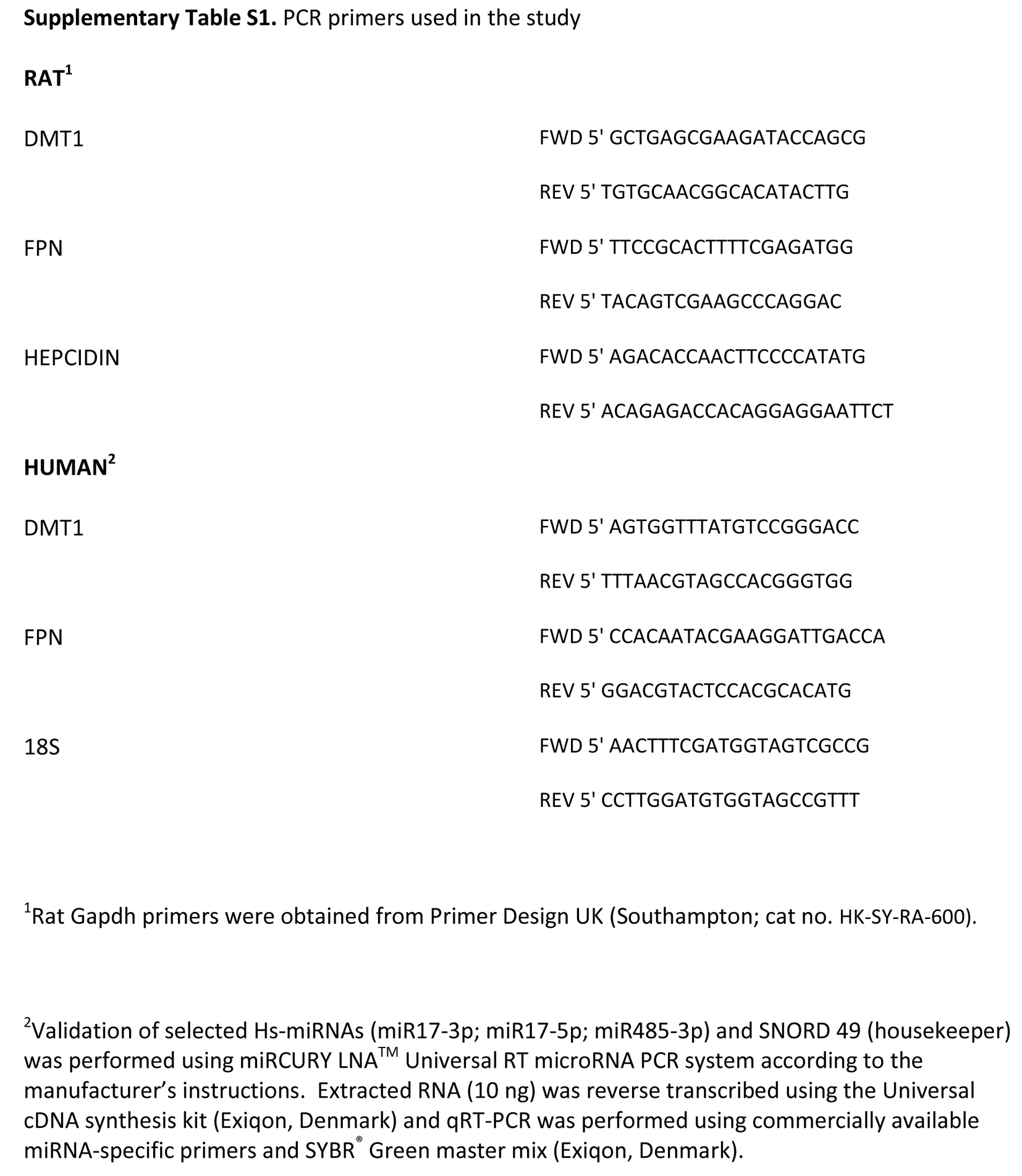

Supplement: Table S1 — PCR primers used in the study. (TIF) [file pone.0102900.s004.tif]

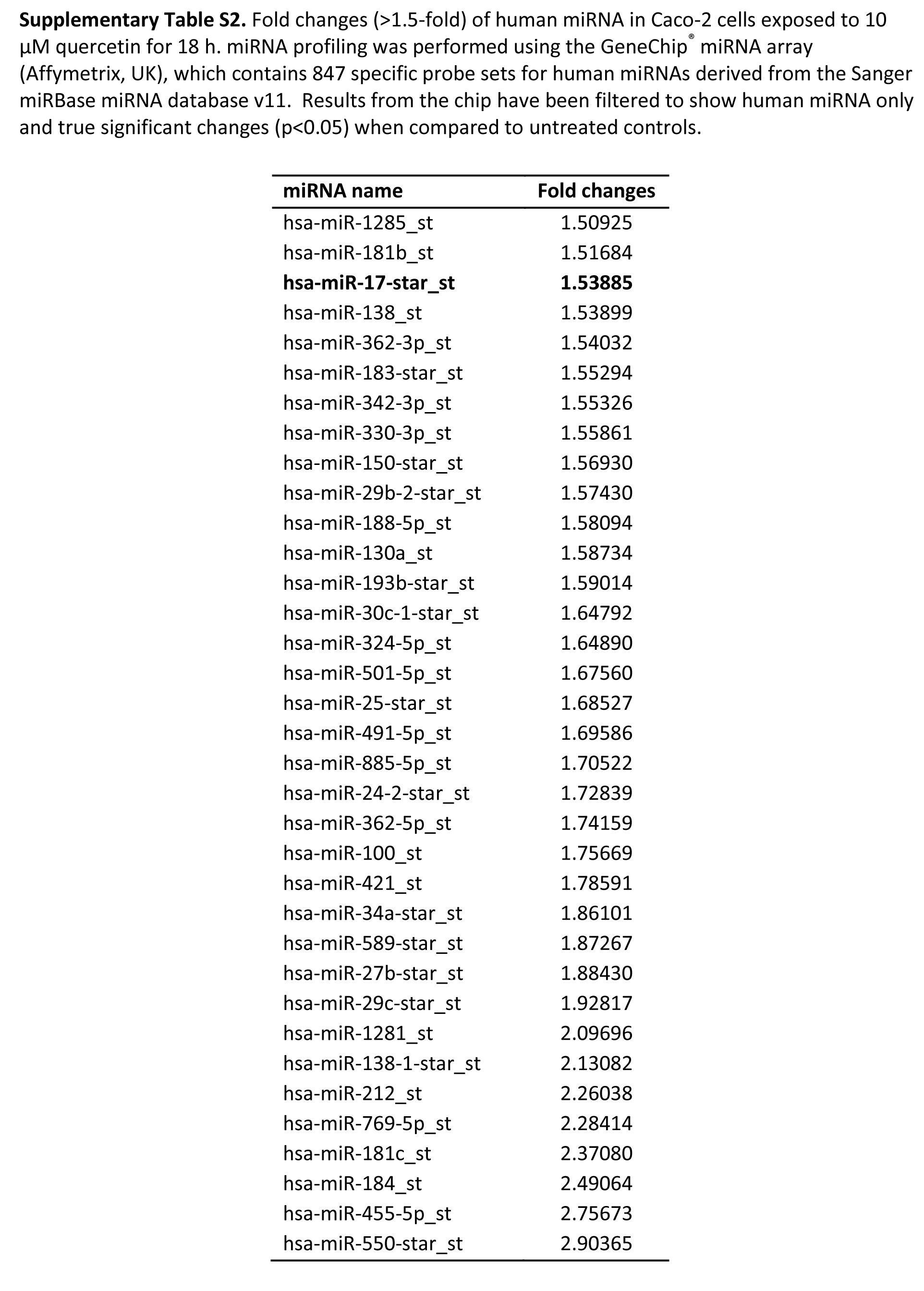

Supplement: Table S2 — Fold changes (>1.5-fold) of human miRNA in Caco-2 cells exposed to 10 µM quercetin for 18 h. miRNA profiling was performed using the GeneChip miRNA array (Affymetrix, UK), which contains 847 specific probe sets for human miRNAs derived from the Sanger miRBase miRNA database v11. Results from the chip have been filtered to show human miRNA only and true significant changes (p<0.05) when compared to untreated controls. (TIF) [file pone.0102900.s005.tif]

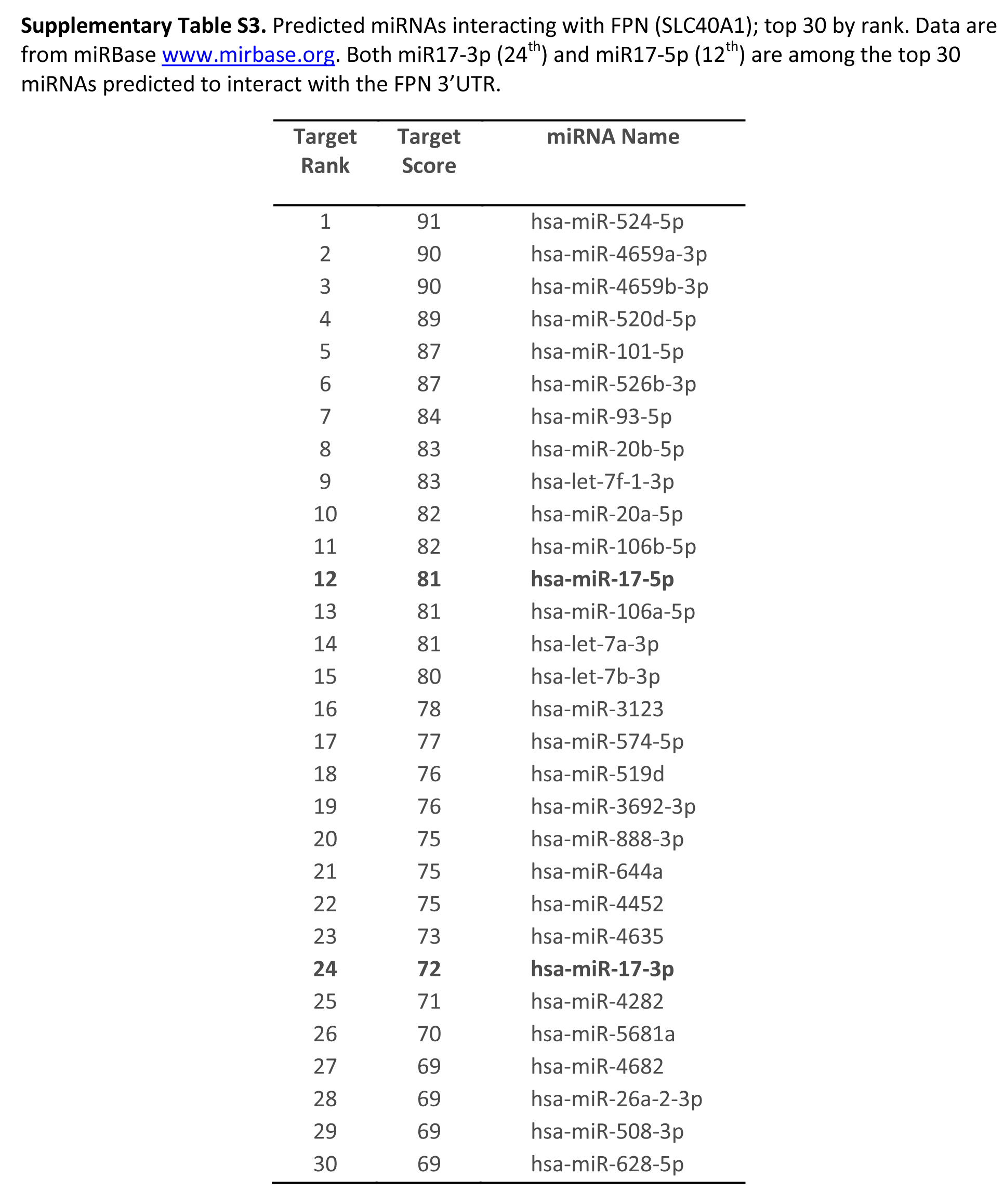

Supplement: Table S3 — Predicted miRNAs interacting with FPN (SLC40A1); top 30 by rank. Data are from miRBase www.mirbase.org. Both miR17-3p (24th) and miR17-5p (12th) are among the top 30 miRNAs predicted to interact with the FPN 3′UTR. (TIF) [file pone.0102900.s006.tif]
